# Supplementary material for: Structures of Native Doublet Microtubules from Trichomonas vaginalis Reveal Parasite-Specific Proteins as Potential Drug Targets
Source: Res Sq. 2024 Sep 2:rs.3.rs-4632384. Preprint. [Version 1] doi: 10.21203/rs.3.rs-4632384/v1 (PMC11398567; doi:10.21203/rs.3.rs-4632384/v1)
Supplement: 1 — Figure S1. Fitted models in cryo-EM densities. Examples of cryo-EM maps with fitted atomic models of MIP and MOP proteins. Figure S2. Docking experiments of β-tubulin and TvFAP40. (a) Atomic model of β-tubulin with putative BZ drug binding site boxed. (b) WT Tv β-tubulin with docked thiabendazole (TBZ), fit into putative binding site. (c) Tv β-tubulin Y168A, P201A mutant with docked TBZ in putative binding site. (d) Atomic model of TvFAP40 with putative IP6 binding site boxed. (e) TvFAP40 binding pocket with docked IP6. (f) TvFAP40 binding pocket with observed IP6. Figure S3. Analysis of TvFAP40 and TvFAP35 and structural homologs. (a) AlphaFold-predicted models of TvFAP35 and TvFAP40 (top) colored by AlphaFold confidence interval (blue more confident, red less confident) and their atomic models (bottom) colored in cyan and magenta respectively. (b) AlphaFold-predicted structures for structural homologs from selected species, colored by AlphaFold confidence interval. (c) Sequence alignment of dimerization and MT binding domain regions from proteins in a and b aligned to TvFAP35, with conserved residues highlighted and those at the active site indicated with arrows. (d and e) α-carbon backbone aligned models from the MT-binding and dimerization domains of the kinked-coiled-coil domains. (f) Conserved proteins from c shown at their locations at the MT-binding interface on 40 TvFAP35. (g-h) Same as f but based on both faces of the dimerization domain. (i) Phylogeny tree including organisms in which FoldSeek identified similar protein structures. Movie S1. overview of Tv-MIPs. Cross sectional view down the Tv-DMT with MIP and MOP densities colored. Model view of all modeled MIPs rotated to show detail and models of TvFAP35 and TvFAP40 in cyan and magenta respectively. Movie S2. TvFAP40 ligand binding pocket. View flying into putative ligand binding site of TvFAP40. Rotations around the ligand binding site with and without the cryo-EM density. [file NIHMSrs4632384v1-supplement-1.pdf]

Supplementary Table 1 Cryo-EM data collection

	WT *Tv*-DMT 16 nm repeat (EMD-XXXXX) (PDB XXXX)	WT *Tv*-DMT 48 nm repeat (EMD- XXXXX) (PDB XXXX)	WT *Tv*-DMT 96 nm repeat (EMD- XXXXX) (PDB XXXX)
**Data collection and processing**			
Magnification	81,000	81,000	81,000
Voltage (kV)	300	300	300
Electron exposure (e–/Å^2)	45	45	45
Defocus range (μm)	-1.5 to -2.5	-1.5 to -2.5	-1.5 to -2.5
Pixel size (Å)	1.1	1.1	1.1
Symmetry imposed	C1	C1	C1
particle images (no.)	425,317	148,707	76,082
Map resolution (Å)	3.8	4.2	4.4
FSC threshold	0.143	0.143	0.143
Repeat unit (nm)	16	48	96
Symmetry imposed	C1	C1	C1

Supplementary Table 2 MIPS and MOPS

	Location	Protein	Uniprot ID	Copy number in 96 nm repeat	Length (residues)	Modeled residues	C. reinhardtii orthomolog	T.thermophila ortholog	Human ortholog
1	Tubulin	α tubulin	A2E8B1	276	452	1-439	α tubulin	α tubulin	α tubulin
2	Tubulin	β tubulin	A2DC16	276	447	1-428	β tubulin	β tubulin	β tubulin
3	A-tubule	Rib72	A2GCC1	12	595	65-595	Rib72	Rib72	EFHC1/2
4	B-tubule	FAP45	A2ETR1	2	465	1-107, 108-465	FAP45	CFAP45	FAP45
5	B-tubule	FAP52	A2FVE3	6	605	1-67, 86-776	FAP52	CFAP52	FAP52

6	Inner Junction	PACRGB	A2EJQ5	6	241	18-235	PACRG	PACRG	PACRG
7	A-tubule	FAP21	A2F5C9	2	386	321-386	FAP21	FAP21	FAP21
8	Inner Junction	PACRGA	A2DAX1	6	236	42-233	PACRG	PACRG	PACRG
9	A-tubule	FAP53	A2G223	2	482	1-224, 238-470	FAP53	CFAP53	FAP53
10	A-tubule	TvFAP12	A2F1C6	2	108	1-108	_	_	_
11	B-tubule	CCDC173	A2EFC9	2	455	33-355, 374-455	FAP210	CCDC173	FAP210
12	A-tubule	FAP115	A2F0U9	3	927	1-200, 227-350, 460-628, 713-927	FAP115	CFAP115	_
13	A-tubule	FAP67	A2E829	2	375	1-375	FAP67	CFAP67A	NME7
14	B-tubule	Tv-FAP35	A2DUL4	6	306	8-19, 27-306	_	_	_
15	B-tubule	Tv-FAP40	A2DSS2	6	377	1-377	_	_	_
16	Ribbon	Rib43a	A2FZ95	2	383	1-357	Rib43a	Rib43a	RIBC2
17	B-tubule	FAP77	A2FTW1	6	283	71-283	FAP77	CFAP77	FAP77
18	A-tubule	FAP127	A2FH94	2	490	1-490	FAP127	CFAP127	MNS1
19	B-tubule	Enkurin	A2EMB8	6	241	1-241	FAP106	A8I9E8	ENKUR
20	Inner Junction	FAP20	A2EAE1	12	194	1-184	FAP20	CFAP20	FAP20
21	N-DRC	DRC4	A2FWB4	1	512	315-493	DRC4	DRC4	DRC4
22	N-DRC	DRC2	A2GIM1	1	461	251-339, 340-411	DRC2	DRC2	DRC2
23	N-DRC	DRC1	A2DEK4	1	633	292-370, 522-633	DRC1	DRC1	DRC1
24	External coiled coils	CFAP58	A2FE28	1	870	480-726, 727-870	FAP189	CFAP58	FAP58
25	Radial Spoke Base	FAP253	A2DGT8	1	396	200-270, 313-374	FAP253	CFAP253	IQUB
26	Radial Spoke Base	CCDC96	A2DTI0	1	363	214-315, 316-361	FAP184	CCDC96	CCDC96
27	External coiled coils	CCDC39	A2EKX3	1	998	3-37, 58-299	FAP59	CCDC39	CCDC39
28	External coiled coils	CCDC40	A2DSS7	1	889	77-95, 105-311	FAP172	CCDC40	CCDC40
29	N-DRC/radial spoke	FAP91	A2F6E6	1	602	305-660	FAP91	CFAP91	FAP91

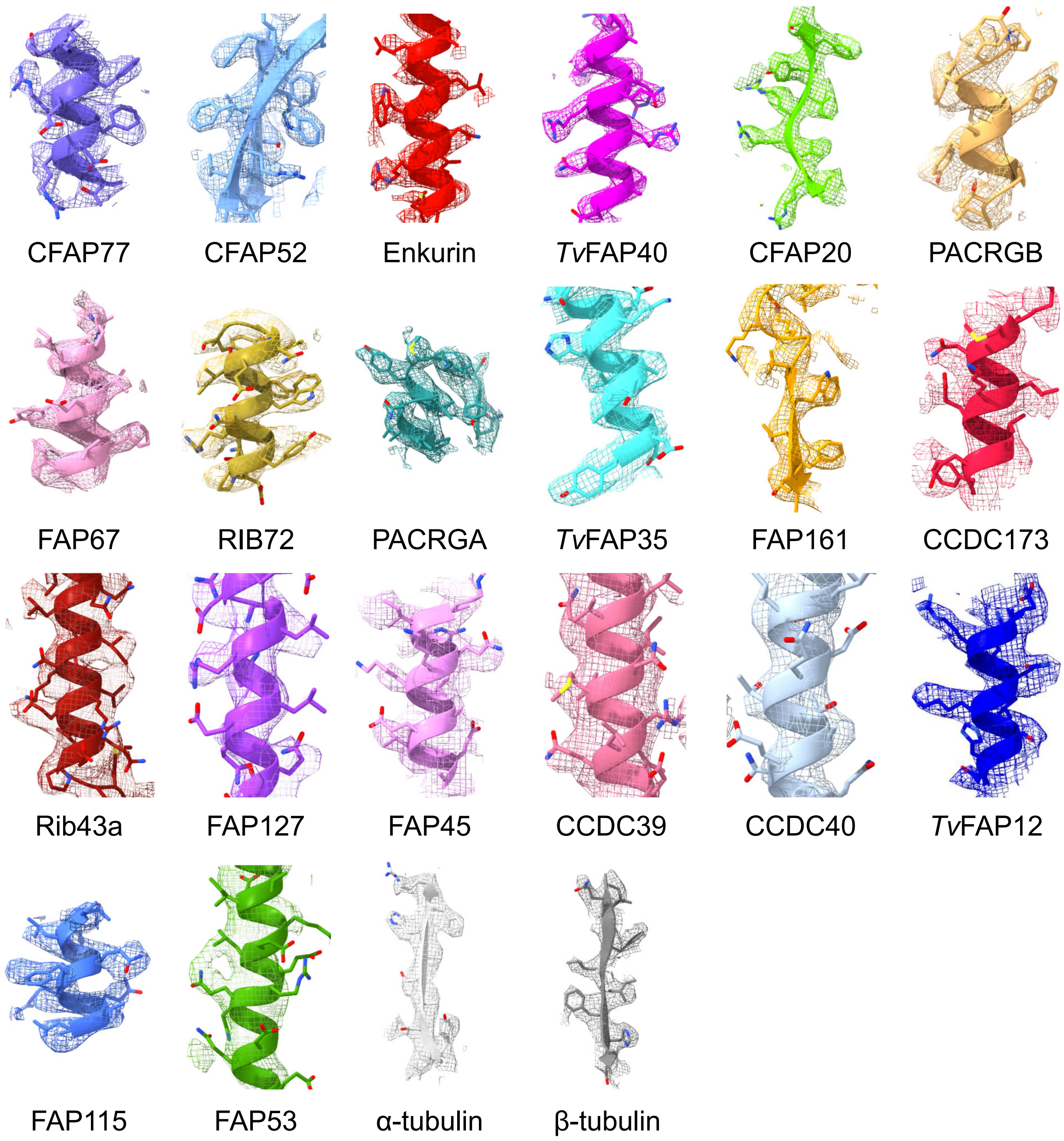

Figure S1. Fitted models in cryo-EM densities. Examples of cryo-EM maps with fitted atomic models of MIP and MOP proteins.

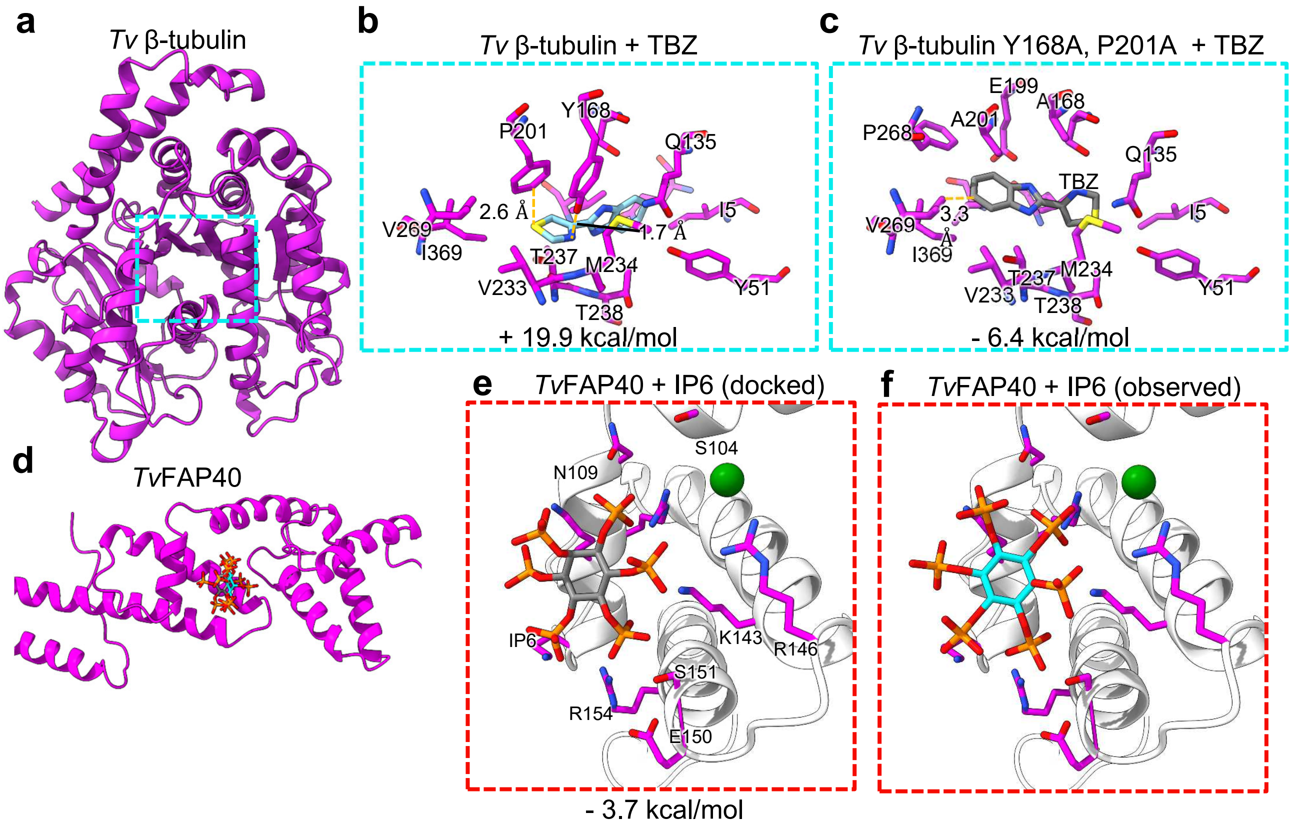

Figure S2. Docking experiments of β-tubulin and *Tv*FAP40. (**a**) Atomic model of β-tubulin with putative BZ drug binding site boxed. **(b)** WT *Tv* β-tubulin with docked thiabendazole (TBZ), fit into putative binding site. (**c**) *Tv* β-tubulin Y168A, P201A mutant with docked TBZ in putative binding site. (**d**) Atomic model of *Tv*FAP40 with putative IP6 binding site boxed. (**e**) *Tv*FAP40 binding pocket with docked IP6. (**f**) *Tv*FAP40 binding pocket with observed IP6.

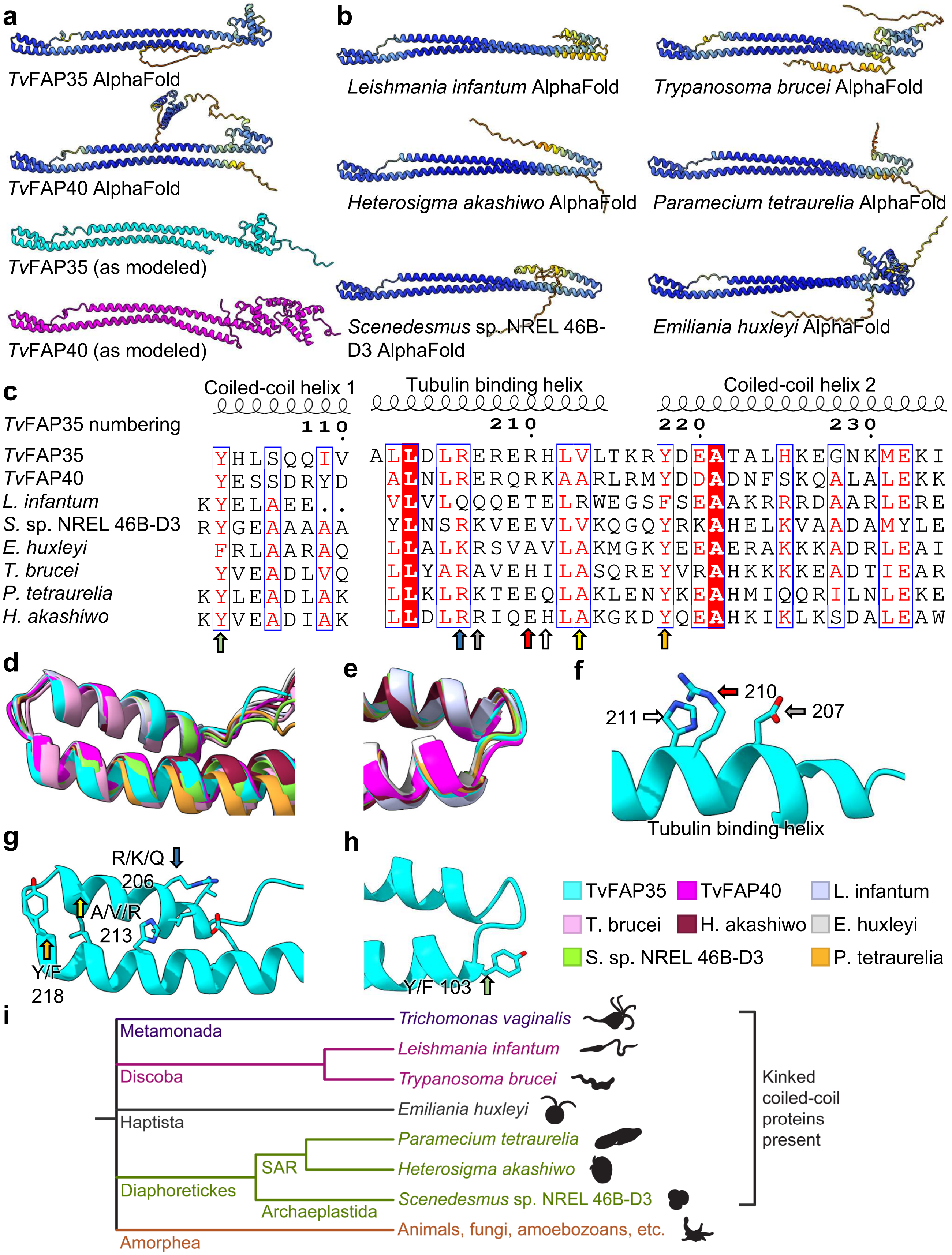

Figure S3. Analysis of *Tv*FAP40 and *Tv*FAP35 and structural homologs. (**a**) AlphaFold-predicted models of *Tv*FAP35 and *Tv*FAP40 (top) colored by AlphaFold confidence interval (blue more confident, red less confident) and their atomic models (bottom) colored in cyan and magenta respectively. **(b)** AlphaFold-predicted structures for structural homologs from selected species, colored by AlphaFold confidence interval. (**c**) Sequence alignment of dimerization and MT binding domain regions from proteins in **a** and **b** aligned to *Tv*FAP35, with conserved residues highlighted and those at the active site indicated with arrows. (**d and e**) α-carbon backbone aligned models from the MT-binding and dimerization domains of the kinked-coiled-coil domains. (**f**) Conserved proteins from **c** shown at their locations at the MT-binding interface on *Tv*FAP35. (**g-h**) Same as **f** but based on both faces of the dimerization domain. (**i**) Phylogeny tree including organisms in which FoldSeek identified similar protein structures.

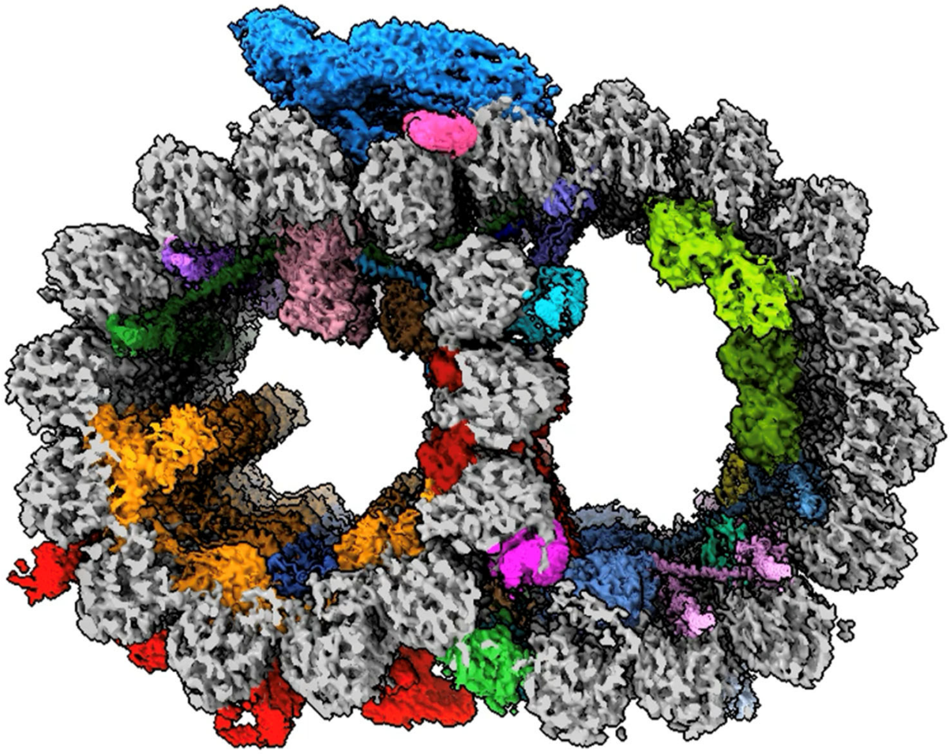

Movie S1. overview of *Tv*-MIPs. Cross sectional view down the *Tv*-DMT with MIP and MOP densities colored. Model view of all modeled MIPs rotated to show detail and models of *Tv*FAP35 and *Tv*FAP40 in cyan and magenta respectively.

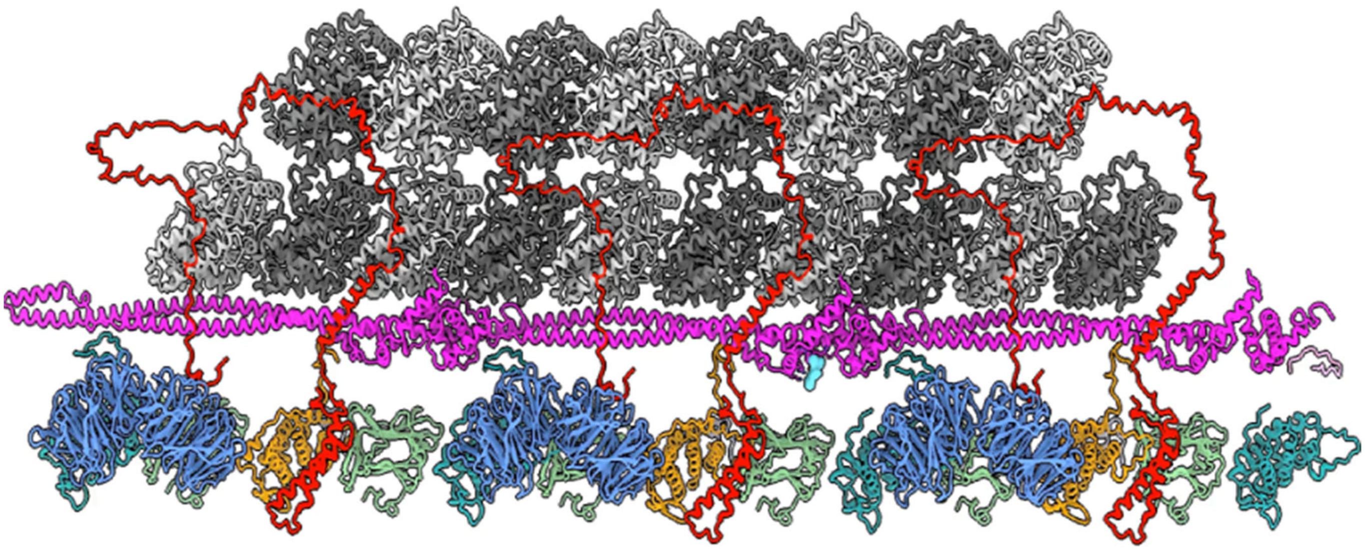

Movie S2. *Tv*FAP40 ligand binding pocket. View flying into putative ligand binding site of *Tv*FAP40. Rotations around the ligand binding site with and without the cryo-EM density.
